# Caregiver burden and associated factors amongst carers of women with advanced breast cancer attending a radiation oncology clinic in Nigeria

**DOI:** 10.4102/phcfm.v13i1.2812

**Published:** 2021-06-15

**Authors:** Ikechi E. Jite, Adedotun A. Adetunji, Ayorinde M. Folasire, Joshua O. Akinyemi, Segun Bello

**Affiliations:** 1 Department of Family Medicine, University College Hospital, Ibadan, Nigeria; 2 Department of Radiation Oncology, Faculty of Clinical Sciences, University of Ibadan, Ibadan, Nigeria; 3 Department of Epidemiology and Medical Statistics, Faculty of Public Health, University of Ibadan, Ibadan, Nigeria

**Keywords:** family caregivers, caregiver burden, Zarit Burden Interview, advanced breast cancer, Nigeria

## Abstract

**Background:**

The responsibility of caring for patients with advanced cancer in sub-Saharan Africa is mostly shouldered by family members because of paucity of institutional facilities. There is a growing concern that the number of women needing treatment for advanced breast cancer is rising at an unprecedented rate in Nigeria.

**Aim:**

To assess the caregiver burden and its associated factors amongst family caregivers of women with advanced breast cancer.

**Setting:**

The study was conducted at the radiation oncology clinic of the University College Hospital, Ibadan, Nigeria.

**Methods:**

A cross-sectional descriptive study was conducted amongst 157 eligible family caregivers of women with advanced breast cancer. The family caregivers completed an interviewer-administered questionnaire, which included the socio-demographic data, the caregiving process and the Zarit Burden Interview (ZBI). Logistic regression was used to identify factors, and ethical approval was obtained.

**Results:**

Over half (53%) of the respondents were males with spousal caregivers dominantly constituting 27.4% of all respondents, closely followed by daughters (25.5%) of the care recipients. The mean ZBI score was 29.84 ± 13.9. Most (72%) of the caregivers experienced burden. Factors associated with caregiver burden were previous hospitalisation of the care recipient (odds ratio [OR] = 3.74, confidence interval [CI]: 1.67 to 8.38) and perceived dysfunction in patients activities of daily living (OR = 2.57, CI: 1.14 to 5.78).

**Conclusion:**

Family caregivers of women with advanced breast cancer experience burden of care. Recognition of this vulnerable population and the care recipient as a dyad is a sine qua non in mitigating the burden associated with their caregiving role.

## Introduction

Breast cancer is the commonest form of cancer in women globally with over two million cases in 2018.^1^ Breast cancer is now common in most part of the world.^2^ The widespread belief that breast and other cancers are rare in low-income regions such as Africa is a myth. In these low-income settings, the case fatality rate is high (in comparison to high-resource countries) as women with breast cancer either present with large, advanced tumours or do not present until the disease is at an incurable stage.^3^ Metastatic or advanced breast cancer is the presence of disease at distant sites such as bone, liver or lung. It is not treatable by primary surgery and is currently considered incurable.^4^

Increasing incidence rates, longer survival times, reductions of stays in acute care settings and shifting of treatment towards ambulatory care have transformed cancer into a continuous care problem with consequent increased responsibilities on family members for both the physical and emotional care of patients with cancer.^5^

In Nigeria and other West-African sub-regions, hospice and palliative care facilities to treat cancer patients are few.^6^ The rising incidence of cancer and the paucity of institutional facilities and specialist manpower therefore imply that the burden of caring for patients with cancer is borne largely by family caregivers with little or no public assistance.^6,7^ Like in most sub-Saharan African countries, the increasingly fragile extended family system in Nigeria has to bear the major burden of chronic medical illness, because there are no national social welfare provisions.^8^ Many patients diagnosed with advanced breast cancer will eventually require support from a family caregiver. The needs of family caregivers often go unnoticed. Family caregivers are not the ‘identified patients’ (they have been called the ‘hidden patients’^9^), and formal care is usually predicated on the identified needs and medical condition of the care recipient.

Whilst a growing body of literature discusses caregiving of the elderly with Alzheimer’s, dementia, mental illness, brain injury and mental retardation, research on informal caregiving to cancer patients is sparse.^10,11^ The burden of care has not been well studied amongst women with advanced breast cancer in Nigeria. It is not known whether the major determinants of family caregiver burden in this study environment are intrinsic or extrinsic to the breast cancer caregiver. It is also not clear from a review of available literature whether the severity of burden borne by family caregivers of breast cancer patients in University College Hospital (UCH), Nigeria corresponds with clinical symptoms and signs in these carers. This study, therefore, sought to assess the burden of the caregiving role on the family caregivers of women with advanced breast cancer and identify factors associated with family caregiver burden amongst participants attending the radiation oncology clinic of the University College Hospital, Ibadan, Nigeria.

## Methods

### Study setting and participants

The study was carried out at the outpatient clinic of the Radiation Oncology Department in the UCH, Ibadan, Nigeria. The clinic is one of the eight established radiotherapy centres in Nigeria. It serves as a referral centre for cancer patients that require radiation therapy from units within the UCH clinics and hospitals outside (across Nigeria and the West African sub-region).

On an annual basis, about 200–300 patients with breast cancer are treated. The study population consisted of 157 adult informal caregivers of women with advanced breast cancer, who were attendees at the clinic over a 3-month period between August 2012 and October 2012.

### Study design

A descriptive cross-sectional survey design was adopted for this study, which identified informal or family caregivers accompanying the women with advanced breast cancer. To be eligible, caregivers had to be: (1) recognised as a significant treatment supporter by a registered patient with advanced breast cancer; (2) aged 18 years and above as at last birthday; (3) personally involved in at least three domains of care such as physical, emotional and financial for a woman with a medical diagnosis of advanced breast cancer; (4) involved with informal caregiving for at least 3 months to the care recipient; and (5) willing to participate as evidenced by a written informed consent. Pregnant women and other caregivers who were paid for services rendered and those unwilling to consent were excluded from the study.

A minimum sample size of 157 was required for the study. This was estimated from the formula for obtaining single mean (${\text{Z}}_{\alpha }{}^{2}{ð}^{2}/{\text{e}}^{2}$)^12^ using a standard deviation (ð) of Zarit burden interview (ZBI) of 12.8 obtained from a similar study conducted by Yusuf et al.^6^ and an estimate of *e* = 2. To achieve a sample size of 157, eligible participants were selected using a simple random sampling. There were four clinic days in a week. This gave a total of 16 clinic days per month. Thus, for a 3-month study period, there were 48 days to interview the eligible participants. Number of participants per day over a three month study period was derived as follows:

$$\frac{\text{Sample size}}{\text{number of clinic days}}=\frac{157}{48}=3$$ [Eqn 1]

Thus, a minimum of three questionnaires were targeted to be administered per clinic day.

The family caregivers accompanying patients on each clinic day were serially registered. This register served as the sampling frame for the simple random selection.

Considering the sampling frame, every eighth caregiver was selected to meet up a total of three respondents per day. The numbers were generated using the total list of caregivers by the random number function in Microsoft Excel. Caregivers whose serial numbers on the register were amongst those generated by the computer were selected for participation in the study. For a randomly selected family caregiver not willing to participate, the next family caregiver with a number on the list of eighth number generated by the computer was approached. Once the intake capacity was maximised, recruitment was stopped for the day.

### Data collection

Data collection was carried out by means of an interviewer-administered questionnaire. Each questionnaire consisted of three sections. Section A sought information on the socio-demographic characteristics of the respondents.

Section B was designed by the researcher and face-validated by three senior colleagues (consultant family physicians) to limit ambiguity and ensure simplicity and clarity. This section sought information on the caregiving process. Section C contained the ZBI. The ZBI is a 22-item self-report inventory developed originally to assess burden in relatives of patients with dementia but it has grown in popularity (amongst multiple populations) and has also been used to assess burden in relatives of patients with cancer even in Nigeria.^6,13,14^It assesses a caregiver’s health, psychological well-being, finances, social life, stigma and relationship to patient.^13^ The caregiver indicates how much discomfort the occurrence of particular items causes and rates each item on a 5-point rating scale ranging from 0 (never) to 4 (nearly always). Responses are summed to generate a total burden score. The summated score (of the 22 items) range from 0 to 88 points with higher scores indicating greater burden. Levels of burden are categorised as no burden (0–20), mild burden (21–40), moderate burden (41–60) and severe burden (61–88). The ZBI is widely used, and data obtained from various studies demonstrate good internal consistency, with Cronbach’s alphas above 0.80.^15^ The ZBI has been validated (test–retest reliability and face validity established) in Nigeria where it has previously been used.^16^

The questionnaire was translated to the predominant local language (Yoruba) and back-translated to English to ensure that each question conveyed the expected meaning. The research assistant engaged was also proficient in both English and Yoruba. A reliability test carried out on the pre-tested data showed a Cronbach’s alpha of 0.87 indicating good internal consistency for the study instrument.

### Data analysis

For this study, primary outcome or dependent variable included the ZBI score (discrete variable) as a measure of the severity of family caregiver burden perceived by the participant. The independent variables included the socio-demographic characteristics of the caregiver such as age of caregiver (in years) as an estimate of participant’s maturity and employment status of caregiver (dichotomous variable) as a marker of participant’s extra-crisis responsibilities. Some other independent variables in this study include caregiver’s relationship to patient (nominal variable) as a marker of social connection, perceived dysfunction in care recipients’ activities of daily living (ADL), duration of caregiving and previous hospitalisation of the care recipient.

Each questionnaire retrieved was coded serially and entered into the computer using Statistical Package for the Social Sciences (SPSS) version 20 for data analysis. The data entered were cleaned and subjected to descriptive (i.e. mean and standard deviation) and inferential (i.e. chi-square) analysis. Statistically significant variables were further subjected to binary logistic regressions to adjust for cofounders and determine possible predictors of the outcome variables. All analyses were carried out at *p* ≤ 0.05 representing the level of significance. Results were summarised and presented in tables and charts.

### Ethical considerations

The joint institutional review board of the University College Hospital Ethics Committee (UI/UCH EC) and the University of Ibadan approved the research (registration number: NHREC/05/01/2008a, assigned number: UI/EC/12/0017). Participants gave written informed consent. The care recipients also gave verbal assent. The serially coded questionnaires were entered into the computer. Only the researcher, data entry clerk and the statistician had access to the data.

## Results

All 157 participants recruited were analysed. The male respondents constituted 53% of the population studied.

The mean age of the study population was 41.6 ± 14.7 years. The majority, 117 (74.5%), of the respondents were married, in monogamous relationships, 98 (83.8%). About four-fifth of the respondents, 122 (77.7%), were employed, whilst 118 (75.2%) respondents lived above the poverty level of $1.25 at the time of this study (as defined by the World Bank^17^). The socio-demographic characteristics of the respondents are shown in Table 1.

**TABLE 1:** Socio-demographic characteristics of the respondents.

Variable	Frequency	%
**Age (years)**		
< 30	39	24.8
30–39	39	24.8
40–49	32	20.4
50–59	23	14.6
≥ 60	24	15.3
**Educational status**		
No formal education	10	6.4
Primary education	17	10.8
Secondary education	49	31.2
Tertiary education	81	51.6
**Marital status**		
Not married	40	25.5
Currently married	117	74.5
**Type of marriage (*N* = 117)**		
Monogamous	98	83.8
Polygamous	19	16.2
**Work status**		
Employed	122	77.7
Unemployed	35	22.3
**Average monthly income regrouped**		
< $1.25	39	24.8
≥ $1.25	118	75.2
**Religion**		
Christianity	111	70.7
Islam	46	29.3
**Tribe regrouped**		
Yoruba	117	74.5
Hausa	3	1.9
Igbo	24	15.3
Others†	13	8.3

*N* = 157.

Age mean = 41.6 ± 14.7.

† , Edo, Urhobo, Igede, Delta, Anang and Kogi.

### Relationship between family caregivers and care recipients

**FIGURE 1:**
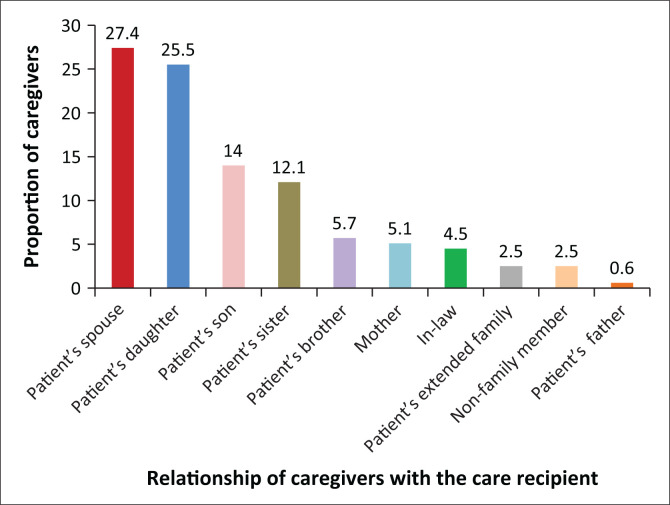
Relationship between family caregivers and care recipients.

The range of family caregivers was mostly relatives. Patients’ spouses had the highest proportion (27.4%) whilst 25.5% were daughters (Figure 1).

### Caregiving-related factors

A total of 51 (32.5%) respondents reported mild dysfunction in ADL, in their care recipients, and 19 (12.1%) respondents reported moderate dysfunction in their care recipients. Respondents who reported severe dysfunction in their care recipient constituted the least proportion of 3 (1.9%).

A total of 56 (35.7%) respondents had been involved in the caregiving of their care recipient for greater than 12 months. In terms of intensity of the caregiving role, 50 (31.8%) respondents reported spending between 7 and 24 h per week and 48 (30.6%) respondents reported putting in greater than 48 h per week; 36 (22.9%) of the respondents did not have support from another (significant others) caregiver. A total of 81 (51.6%) respondents reported previous hospitalisation of their care recipients. The caregiving related factors are shown in Table 2.

**TABLE 2:** Factors related to caregiving.

Variable	Number	%
**Perceived dysfunction in care recipients’ ADL**		
No dysfunction	84	53.5
Mild dysfunction	51	32.5
Moderate dysfunction	19	12.1
Severe dysfunction	3	1.9
**Duration of caregiving (months)**		
< 6	49	31.2
6–11	52	33.1
≥ 12	56	35.7
**Intensity of caregiving (hours/week)**		
Minimal intensity (< 7)	43	27.4
Mild intensity (7–24)	50	31.8
Moderate intensity (25–48)	15	9.6
Severe intensity (> 48)	48	30.6
I am not sure	1	0.6
**Significant others involvement**		
Nobody	36	22.9
Another caregiver	66	42.0
Two or more other caregivers	55	35.0
**Previous hospitalisation**		
Yes	81	51.6
No	76	48.4

*N* = 157.

ADL, activities of daily living.

### Experience of burden by the respondents

The total mean ZBI score from this study was 29.84 ± 13.9. As shown in Table 3, the majority 82 (52.2%) of the respondents experienced mild burden, whilst moderate and severe burden were experienced by 27 (17%) and 4 (2.5%) of the respondents, respectively. Less than a third 44 (28%) of the respondents did not experience burden.

**TABLE 3:** Mean scores of the experience of burden by the respondents.

Overall experience of burden	*N*	%	Mean ZBI score	SD
No burden	44	28.0	13.84	4.36
Mild burden	82	52.2	30.46	5.50
Moderate burden	27	17.2	48.96	6.11
Severe burden	4	2.5	64.00	2.45

**Total**	**157**	**-**	**29.84**	**13.9**

*N* = 157.

SD, standard deviation; ZBI, Zarit burden interview.

### Factors associated with family caregiver burden

A higher proportion, 59 (75%), of the respondents above 40 years experienced burden compared with their counterpart below 40 years with a *p* > 0.05. Gender of the family caregivers was also not significantly associated with burden even though the female caregivers appeared to experience higher level of burden. Level of education of the family caregivers was strongly associated with caregiver burden (*p* = 0.009). Thus, respondents with levels of education below the tertiary level reported greater burden. Other variables like marital status, work status and monthly income did not have a significant statistical relationship with burden.

Although the relationship to the patient did not have a significant association with the experience of burden, daughters of the care recipient had a higher level of burden. Perceived dysfunction in patients’ ADL was significantly associated with caregiver burden (*p* = 0.022) as was previous hospitalisation of the care recipient (*p* = 0.043) and duration of caregiving (*p* = 0.001). Family caregivers with longer duration of caregiving and whose care recipients had dysfunction in ADL and had been previously hospitalised reported higher caregiver burden. Intensity of the caregiving role in terms of hours spent per week caring for the care recipient was not significantly associated with caregiver burden even though respondents who spent greater than 48 h per week in caregiving had a higher burden 40 (81.6%) compared with those who spent less than 48 h per week. Respondents who reported having another caregiver to assist in the caregiving of their care recipient seemed to have a marginally lower burden 87 (71.9%) compared with their counterparts who reported having nobody to assist in the caregiving role. However, the difference was not statistically significant (see Table 4).

**TABLE 4:** Factors associated with caregivers burden.

Variable	Experience of burden						χ^2^	*p*
	No burden (*n* = 44)		Have burden (*n* = 113)		Total (*n* = 157)			
	*n*	%	*n*	%	*n*	%		
**Age at last birthday (years)**								
< 40	24	30.8	54	69.2	78	100.0	0.575	0.448
≥ 40	20	25.0	59	75.0	79	100.0	-	-
**Sex**								
Male	25	30.1	58	69.9	83	100.0	0.381	0.537
Female	19	25.7	55	74.3	74	100.0	-	-
**Educational level**								
< Tertiary education	14	18.4	62	81.6	76	100.0	6.867	0.009*
≥ Tertiary education	30	37.0	51	63.0	81	100.0	-	-
**Marital status**								
Not married	12	30.0	28	70.0	40	100.0	0.104	0.747
Currently married	32	27.4	85	72.6	117	100.0	-	-
**Work status**								
Employed	30	24.6	92	75.4	122	100.0	3.181	0.089
Unemployed	14	40.0	21	60.0	35	100.0	-	-
**Average monthly income**								
< $1.25	9	23.1	30	76.9	39	100.0	0.647	0.421
≥ $1.25	35	29.7	83	70.3	118	100.0	-	-
**Relationship to patient**								
Spouse	12	27.9	31	72.1	43	100.0	1.173	0.759
Son	6	27.3	16	72.7	22	100.0	-	-
Daughter	9	22.5	31	77.5	40	100.0	-	-
Other relations	17	32.7	35	67.3	52	100.0	-	-
**Patients’ ADL dysfunction**								
No dysfunction	30	35.7	54	64.3	84	100.0	5.261	0.022*
Perceived dysfunction	14	19.2	59	80.8	73	100.0	-	-
**Previous hospitalisation of care recipient**								
Yes	17	21.0	64	79.0	81	100.0	4.083	0.043*
No	27	35.5	49	64.5	76	100.0	-	-
**Duration of caregiving (months)**								
< 6	20	40.8	29	59.2	49	100.0	13.414	0.001*
6–11	18	34.6	34	65.4	52	100.0	-	-
≥ 12	6	10.7	50	89.3	56	100.0	-	-
**Intensity of caregiving (hours/week)**								
≤ 48	35	32.4	73	67.6	108	100.0	3.294	0.070
> 48	9	18.4	40	81.6	49	100.0	-	-
**Significant others involvement**	-	-	-	-	-	**100.0**	-	-
Nobody	10	27.8	26	72.2	36	100.0	0.001	1.000
Another caregiver	34	28.1	87	71.9	121	100.0	-	-

ADL, activities of daily living.

* , Statistically significant (*p* < 0.05).

### Predictors of family caregiver burden

In order to identify the principal determinants or predictors of caregiver burden, a logistic regression model (Table 5) was developed with those variables that were significantly related to caregiver burden as determined by the bivariate analysis. The result showed that variables that retained their significant association with caregiver burden were previous hospitalisation of the care recipient (*p* = 0.001) and perceived dysfunction in patients ADL (*p* = 0.022). Family caregivers whose care recipients had been previously hospitalised are three times more likely to experience burden than those with no previous hospitalisation (OR = 3.74, CI: 1.67 to 8.38), whilst family caregivers who had perceived dysfunction in patients’ ADL were two times more likely to experience burden than those caregivers with no perceived dysfunction in care recipient’s ADL (OR = 2.57, CI: 1.14 to 5.78).

**TABLE 5:** Logistic regression analysis of factors associated with caregiver burden.

Variable	OR	Lower	Upper 95% CI	*p*
Highest educational level ≤ Tertiary education	1.743	0.778	3.904	0.177
Previous hospitalisation Yes	3.742	1.671	8.381	0.001
Perceived ADL dysfunction	2.577	1.148	5.780	0.022
Duration of caregiving ≤ 6 months	0.474	0.216	1.038	0.062

OR, odds ratio; CI, confidence interval; ADL, activities of daily living.

## Discussion

This study established a mean caregiver burden of 29.84 ± 13.9 (using the ZBI, mean score). This was comparable with the mean score of 30.55 ± 19.18 documented by Vahidi et al. who looked at caregiver burden amongst primary caregivers of women with breast cancer in Iran^18^ and the mean ZBI score of 29.16 ± 12.8 reported by Yusuf et al. who looked at caregiver burden amongst family caregivers of patients with various cancer diagnoses in north-western Nigeria.^6^ An observational cohort study of caregivers of patients with advanced chronic diseases (cancer, heart failure [HF] and chronic obstructive pulmonary disease) reported that the level of caregiver burden was similar across different patient diagnoses^19^ and comparable with the level of burden previously documented amongst caregivers of patients with dementia^20^ and terminal cancer.^14^ These findings suggest that caregiver burden may not be disease-specific but may be a universal phenomenon of caring for persons with chronic illnesses.^19^

The male gender constituted a predominant proportion of the family caregivers with most of these having spousal relationship with the care recipient. This is comparable with a longitudinal study of breast cancer patients and their primary caregivers, which showed 55% of the caregivers to be male and the patients spouse or male intimate partners of the care recipient (52%).^14^ This finding is however not consistent with the tradition in sub-Saharan Africa where women are typically the traditional caregivers for family members with chronic illness.^21,22^

Although the informal caregiving landscape is still dominated by women, men are more likely to participate if the illness affects a spouse or mate or if no woman was available to assume the role. Previous studies have established the central position occupied by spouses in giving emotional support and providing physical assistance to the woman with breast cancer.^14,23,24^ A study in Canada aimed at describing the psychosocial impact on caregivers of caring for women with advanced breast cancer identified the adult offspring, most often the daughter, as the primary caregiver if the spouse was not.^25^ This corroborate the findings from this study and that reported by Grunfeld et al.,^14^ which showed that the highest proportion of caregivers were spouses followed closely by the daughters of these breast cancer patients. In sub-Saharan Africa, offsprings constitute the primary caregivers of their sick parents even though the sick parent has a spouse.^6^ In spite of the fact that these adult offsprings would also be dealing with issues involving their family of procreation, there is a high cultural expectation for adult offsprings to provide social supports for their aged parents.^7^ The predominance of male partners in the caregiving role in this study, which is contrary to cultural norm in this environment, may be suggestive of a moral and marital obligation to a spouse in a health crisis situation.

The mean age of 41.6 ± 14.7 in this study showed that the majority of the family caregivers are in the prime of life, and these caregivers are likely to be the spouses of these women, especially because there are a higher proportion of young females with breast cancer in our environment than in developed countries.^26^ The implication of this is the loss of productive man-hours as caregiving takes away time that may otherwise be spent in paid employment.^6,27^ The gender and age of the caregivers were however not statistically significantly associated with the perception of burden in contrast to findings by Vahidi et al. that showed a statistically significant correlation between the male gender and age and the caregiver burden.^18^ It is noteworthy however that caregivers who were daughters to the care recipient in this study averagely reported a higher burden of care than caregivers who were in a spousal or male offspring relationship with the care recipient even though this relationship did not reach statistical significance. Reversal of roles, another conflict as a result of the caregiving, has been given as possible explanation for the higher level of burden reported by daughters of the care recipients.^10^ The caregiver daughter of an elderly parent, for example, is forced to deal with the major change in the traditional role of her parent (mother) as caretaker. Now, the role is reversed, and the caretaker has become the care receiver. Consequently, the parent–child relationship changes.^10^ This shift becomes particularly difficult when the parent requires help in self-care such as bathing, dressing, feeding and often toileting.^28^ In contrast to the finding of this study, Yusuf et al. found high level of burden to be significantly associated with care by a son.^6^

Of all the other socio-demographic variables in this study, only the educational level of the family caregiver was significantly associated with caregiver burden. A higher proportion of respondents with lower educational level (below tertiary level) reported caregiving burden when compared with respondents with tertiary education. This is consistent with findings by Papastavrou et al. and Vahidi et al., which showed a significant association between lower level of education and experience of burden.^18,29^ Family caregivers with a higher level of education are more likely to be equipped with better coping strategies, relatively better grasp of the disease trajectory, economic power and a wider social support network. They are also more likely to perceive assistance from friends and family to be a stabilising factor.^10^ Caregivers with less education, on the other hand, may feel more threatened by the patient’s illness and are more likely to report the higher burden.^30^

Perceived dysfunction in care recipients’ ADL was significantly associated with expression of caregiver burden in this study. Family caregivers who reported their care recipients had ADL dysfunction experienced a higher level of burden compared with those whose care recipients had no ADL dysfunction. This finding is consistent with that of a previous study, which showed that a higher level of dependency in ADL contribute to increasing the burden level of caregivers.^31^ If the care recipient had more ADL with which they needed help, this was an indicator of more caregiver burden.^32^

Previous hospitalisation of the care recipient was also significantly associated with expressed caregiver burden in this study. This may be explained by the care recipient’s stage of illness. As the disease progresses, care recipients are more likely to report myriads of symptoms and medication-related side effects,^33,34^ which may require hospitalisation. Thus, during the adjuvant treatment phase, the family caregiver may be required to provide tangible assistance to the patient, including transportation to and from treatment centre and emotional support before, during and following the treatment.^10^ Clearly every cycle of hospitalisation and discharge and its attendant demands may portend a period of heightened caregiving burden.

Longer duration of care was also significantly associated with caregiver burden in this study. This however contrasted with findings from a cross-sectional study of 51 caregivers of older persons attending the Geriatric Clinic at an urban hospital in Kuala Lumpur, Malaysia, and that from a cross-sectional study of 200 caregivers of cancer patients attending a tertiary care hospital in Pakistan, both of which showed that shorter duration of caregiving is associated with higher level of burden and longer duration of caregiving is associated with lower level of burden.^35,36^ The incongruence between study’s findings may reflect cultural variations in the different study environments. Our finding, however, may suggest the dwindling of social support with longer duration of caregiving.

The result of the logistic regression model fitted to determine the independent predictors of caregiver burden confirmed that previous hospitalisation and perceived dysfunction in ADL of the care recipients were associated with caregiver burden in this study. The degree of burden experienced by caregivers may depend on disease progression suffered by the care recipient and the perceived stress resulting from caregiving. As the disease progresses, care recipients are more likely to experience multiple hospitalisations that clearly heightens the burden experienced by the family caregivers. This is because the family caregivers may be required to provide tangible assistance to the care recipient.^10^ ADL and instrumental ADL are frequently used as indicators of the functional status of care-recipients, and they independently affect caregiver burden as documented in previous studies.^37,38,39^ However, care-recipients’ functional decline has been an inconsistent predictor. Some studies suggest that moderate to severe disability affecting basic daily activities in care-recipients is related to high caregiver burden.^37,38,39^ However, other studies have found weak or no association between care-recipients’ functional decline and caregiver burden.^40,41^ The finding of ADL as a significant predictor of caregiver burden in this study may indicate that any decline in basic ADL requires a higher level of caregiver engagement and devotion to managing daily life because of increasing dependence of care-recipients.

## Limitation of the study

Only one hospital was used for the study, which may cast some doubts on the generalisability of the study’s findings. The socio-demographic characteristics of the care recipients were not measured. Thus, there may be more factors associated with caregiving burden that has not been assessed by this study.

Because a cross-sectional design makes it impossible to infer causal effects, a similar study with a longitudinal design is recommended to assess the true predictive value of the factors associated with caregiver burden. Further research which should include socio-demographic variables of the care recipients is needed to identify strategies to offset caregiver burden.

## Conclusion and recommendations

Family caregivers of women with advanced breast cancer experience clinically relevant burden of care, predominantly mild to moderate in severity. Significant predictors of family caregiver burden include previous hospitalisation of the care recipient and perceived dysfunction in care recipients’ ADL. Therefore, training of family caregivers to upgrade their skill-set is a sine qua non in mitigating the burden associated with their caregiving role.

Family physicians being frontline doctors in ready contact with family caregivers should be ready to provide opportunistic interventions and coping strategies for reducing the burden of informal caregiving.

A therapeutic alliance between the oncology specialist and the family physician (shared care) will allow the oncology specialist to refer family caregivers to family physicians periodically for assessment. This will help to identify high level of burden. Integrating palliative care in primary care settings is likely to achieve a better outcome in the patient–caregiver dyad.
